# Nurses' knowledge, barriers and practice in the care of patients with delirium in the intensive care unit in Poland—A cross-sectional study

**DOI:** 10.3389/fpubh.2023.1119526

**Published:** 2023-03-03

**Authors:** Sandra Lange, Wioletta Mȩdrzycka-Da̧browska, Lucyna Tomaszek, Magdalena Wujtewicz, Sabina Krupa

**Affiliations:** ^1^Department of Internal and Pediatric Nursing, Faculty of Health Sciences, Medical University of Gdańsk, Gdańsk, Poland; ^2^Department of Anaesthesiology Nursing and Intensive Care, Faculty of Health Sciences, Medical University of Gdansk, Gdańsk, Poland; ^3^Faculty of Medicine and Health Sciences, Andrzej Frycz Modrzewski Krakow University, Kraków, Poland; ^4^Department of Thoracic Surgery, Institute of Tuberculosis and Lung Diseases, Rabka-Zdrój, Poland; ^5^Department of Anaesthesiology and Intensive Therapy, Faculty of Medicine, Medical University of Gdańsk, Gdańsk, Poland; ^6^Institute of Health Sciences, College of Medical Sciences of the University of Rzeszow, Rzeszow, Poland

**Keywords:** delirium, knowledge, delirium assessment, nursing practice, barriers, ICU, evidence-based nursing practice

## Abstract

**Background:**

Delirium is a cognitive disorder that occurs with high frequency in patients in intensive care units and affects patient outcomes. Despite recommendations for monitoring and assessing delirium in the ICU, studies show that it is still not routinely assessed and often remains undiagnosed or misinterpreted as dementia or depression.

**Aim:**

The aim of this study was (1) to assess nurses' knowledge and clinical practices regarding delirium, (2) to identify the factors associated with nurses' knowledge, and (3) to define barriers to effective control of delirium.

**Methods:**

A cross-sectional study was conducted among 371 ICU nurses in Poland.

**Results:**

53.1% of nurses had never been educated on delirium control resulting in a deficit in knowledge of delirium symptoms, risk factors and complications associated with delirium in ICU patients. Master's degree in nursing (vs. Registered nurses + Bachelor's), female gender, and working in university hospital (vs. other) were positively correlated with nurse's knowledge, while age had a negative impact on knowledge. Delirium is a marginalized state in ICU patients, only 16.4% of nurses assessed delirium routinely and 35.8% assessed delirium occasionally, rarely using validated scales. Barriers to effective delirium control were primarily the lack of a requirement to assess delirium, the difficulty of assessing delirium in intubated patients and nurses' lack of confidence in their ability to use delirium assessment tools.

**Conclusions:**

There is an urgent need to educate nurses about delirium and to make delirium assessment obligatory in clinical practice. The area of change should also include a hospital policy on delirium monitoring and management. The study was registered on ClinicalTrials.gov (NCT05384964).

## 1. Introduction

Delirium is defined as an acute cognitive disorder accompanied by fluctuations in mental status and disturbances in attention and consciousness (1, 2). The exact cause of delirium is unclear, but the etiology is thought to be multifactorial (3). Of the 28 risk factors defined by Wu et al. pain, use of physical coercion, respiratory disease, sleep deprivation and surgery were considered the most modifiable. In contrast, non-modifiable risk factors included age and gender (4). According to studies the prevalence of delirium ranges from 32 to 80% (5–7). This complication adversely affects patient outcomes, increasing ICU length of stay, mortality and causing the development of cognitive impairment after ICU hospitalization (8–11). There are three subtypes of delirium: hypoactive, hyperactive, and mixed. In the ICU, the most common form is hypoactive, which is characterized by reduced motor activity, reduced alertness and sleepiness (7, 12). However, due to its silent clinical presentation, this form is the least identified by clinicians (13). Hyperactive delirium is manifested by an increased number of spontaneous movements that are aimless, uncontrolled, and ineffective. The mixed form occurs when the patient's condition oscillates between hyperactive and hypoactive delirium (13).

Prevention and early detection of delirium is key to improving ICU patient safety and provides an opportunity to implement appropriate interventions to reduce its adverse effects (14). Delirium, as a medical diagnosis, often remains unrecognized or misinterpreted by medical staff in critically ill patients, despite the availability of validated tools for the assessment of delirium, such as: Cognitive Test for Delirium, abbreviated Cognitive Test for Delirium, Confusion Assessment Method for the Intensive Care Unit (CAM-ICU), Intensive Care Delirium Screening Checklist (ICDSC), Neelon and Champagne Confusion Scale (NEECHAM), and the Delirium Detection Score (DDS), Nursing Delirium Screening Scale (NuDESC) (12).

Clinical Practice Guidelines for the Prevention and Management of Pain, Agitation/Sedation, Delirium, Immobility, and Sleep Disruption in Adult Patients in the ICU (PADIS) in Adult Patients in the ICU and National Institute for Health and Care Excellence (NICE) recommend routine screening for delirium in intensive care unit patients using validated tools (15, 16). Unfortunately, researchers report a lack of adherence to these recommendations in clinical practice both worldwide (17) and in Poland (18). A study conducted in 2016 by Kotfis et al. provides evidence that the problem of delirium is ignored among Polish patients hospitalized in the ICU—only 11.9% of wards declared that they monitor this adverse condition (18). In contrast, the results of a study reported by Krupa et al., conducted among a group of 45 nurses in a cardiac intensive care unit, suggest that nurses lack knowledge of the factors that contribute to the development of delirium, are not able to communicate with such patients and, above all, do not know the consequences of the actions they take (19). Unfortunately, gaps in knowledge regarding delirium control in ICUs are not only the domain of Polish nurses. Similar problems are noted by researchers among nurses employed in ICUs, e.g., in the United Kingdom, Australia or Jordan (20–22). Among the barriers to good clinical practice in delirium are the following: knowledge deficit, lack of organizational and management support, misconception that tools are complex, difficulty in assessing intubated and sedated patients, and time-consuming (23).

To our knowledge, there are no data available on the knowledge and actual practices of Polish ICU nurses regarding delirium assessment and potential barriers to delirium assessment. An assessment of the level of knowledge of ICU nurses on delirium may indicate an area of possible gaps in education and implementation of educational programs for ICU staff. In turn, identification of barriers to delirium assessment, may help to implement corrective processes to improve delirium practices in ICUs.

### 1.1. Aims

The aim of this study was (1) to assess nurses' knowledge and clinical practices regarding delirium, (2) to identify the factors associated with nurses' knowledge, and (3) to define barriers to effective control of delirium.

## 2. Methods

### 2.1. Study design

This cross-sectional study was conducted in intensive care units in Poland. The study was registered on ClinicalTrials.gov (NCT05384964).

### 2.2. Participants selection

The target population of the study was nursing staff working in adult intensive care units in Poland. Nurses of non-Polish nationality and those working in neonatal intensive care units (NICUs) were excluded from the study.

### 2.3. Research tools

The survey questionnaire aimed at ICU nurses included: socio-demographic data and two questionnaires: Nurses' Knowledge of Delirium- created by Hare et al. (24), and Nursing Practices and Perceptions Toward Delirium in the Intensive Care Unit—developed by Devlin et al. (25). The original questionnaires were written in English and were translated into Polish according to the Translation, Review, Adjudication, Pretest and Documentation (TRAPD) procedure (26). Minor wording changes have been made in both versions to improve relevance and adapt to Polish needs/realities. The authors of the original questionnaires have agreed to their use.

The questionnaire Nurses' Knowledge of Delirium consists of two parts. In the first, participants indicated the correct answer to questions related to the definition of delirium and the tools used to detect each state. In the second part, participants had to answer “agree,” “disagree,” or “don't know” to a series of 28 statements. Fourteen of these statements related to delirium, its presentation and management, and 14 statements related to delirium risk factors. It should be noted that this questionnaire, according to information from one of its authors, has been used in various countries around the world and has been translated into nine languages other than English. A questionnaire sheet with the correct answers was also obtained from the authors of the original version (24). The scores, which were the sum of the correct answers to the questions in the nurses' knowledge area, ranged from 0 to 37 points.

The Questionnaire Nursing Practices and Perceptions Toward Delirium in the Intensive Care Unit consists of two parts. In the first part, participants indicated answers to questions about sedation and delirium assessment. The questions then focused on practices/perceptions toward delirium and its assessment, including identification of potential barriers to delirium assessment. The questionnaire was developed through a deliberate series of steps that included item generation and construction, followed by pilot testing and refinement (25).

### 2.4. Data collection

Data collection took place between May and August 2022. Due to the ongoing COVID-19 pandemic, at the initial stage the survey questionnaire was distributed electronically *via* the website of the Polish scientific society of anesthesia and ICU nurses, social media (Email, WhatsApp, Facebook). Then, after obtaining approvals from hospital heads/directors, the questionnaires were hand-delivered to ICU ward nurses for distribution to nurses. Nurses who declared that they had completed the questionnaire online were informed not to complete it again. The completed sheets were compacted into sealed envelopes and collected from the ward nurses.

### 2.5. Ethical considerations

The study was approved by the Independent Bioethics Committee for Scientific Research of the Medical University of Gdansk (Approval Number: NKBBN/267/2022).

### 2.6. Statistical analysis

Results were expressed as absolute numbers and percentages (categorical variable) or mean, median, upper, and lower quartile (continuous date). To assess associations between categorical variables the Chi-square test was used. Intergroup differences for continuous data were estimated by Mann-Whitney test. The Shapiro-Wilk test was used to detect departures from normality. Spearman's correlation coefficient (rho) was used to measure the dependent relationship between two continuous variables. It was interpreted as negligible (<0.1), weak (0.1–0.39), moderate (0.4–0.69), strong 0.7–0.89, and very strong (0.9–1.0) (27).

Multivariable linear regression models were calculated to find the relationships between the nurse's knowledge and independent variables (gender, age, job seniority, education level, type of hospital, number of beds in an ICU wards). Independent variables with the *p*-value ≤ 0.1 in simple linear regression models were selected introduced into the forward stepwise regression (equal probability value for entry and removal was 0.05). The assumptions for calculating multiple regression were met (a linear relationship between the dependent variable and each of the independent variables, no multicollinearity—the Variance Inflation Factor <1.5, homoscedasticity—White test *p* > 0.05, multivariate normality—Shapiro–Wilk test *p* > 0.05) (28). The results of all multivariable regression models were presented as standardized regression coefficients (ß) and their 95% confidence intervals (CI), partial *R*^2^.

The statistical analyses were conducted using STATISTICA v.13.3. [TIBCO Software Inc. (2017), Krakow, Poland]. A *p*-value < 0.05 were statistically significant.

## 3. Results

### 3.1. Characteristics of participants

A total of 382 questionnaires were collected. One hundred and three responders completed the questionnaire electronically and 279 completed the questionnaire on paper. Due to incomplete questionnaires, 11 questionnaires were rejected. Ultimately, 371 questionnaires were included in the study. Table 1 shows the sociodemographic characteristics of subjects. The analysis included survey data of 324 female nurses [median aged 42 (32; 50) years] and 47 male nurses [median aged 36 (30; 44) years] caring for the patients in the ICU. Median job seniority of the study subjects was 11 years. The vast majority of responders had a master's degree in nursing (57.9%). Approximately 40% (*n* = 144) of the nursing staff declared that they had completed both qualification and specialization training program in “Anesthesiology nursing and intensive care”. Most responders were employed in a university hospital (47.7%), and in the Pomorskie (*n* = 161; 43.4%) and Podkarpackie (*n* = 106; 28.6%) provinces. The median number of beds in the ICU was 10. Twelve-hour shifts were most frequently reported by staff working in the ICU (75.2%).

**Table 1:** Sociodemographic characteristics of subjects.

**Parameter**		
Age (years)		41 [32; 50]
Job seniority (years)		11 (5,20)
Gender *n* (%)	Female	324 (87.3)
	Male	47 (12.7)
Education, *n* (%)	Registered nurse	50 (13.5)
	Bachelor's in nursing	106 (28.6)
	Master of science in nursing	215 (57.9)
Type of postgraduate education program in “Anesthesiology nursing and intensive care”, *n* (%)	Specialization program—completed	224 (60.4)
	Specialization program—during training	69 (18.6)
	Qualification training program—completed	197 (53.1)
	Qualification training program—during training	35 (9.4)
	Other training program—completed	34 (9.2)
Type of hospital, *n* (%)	University hospital	177 (47.7)
	Other: e.g., municipal, provincial	194 (52.3)
Number of beds in wards of the ICU		10 (8,12)
Shift length	8-h shifts	40 (10.8)
	12-h shifts	279 (75.2)
	24-h shifts	52 (14.0)

Results presented as absolute numbers (percentages) or median [upper and lower quartile]; ICU, Intensive Care Unit.

It is worth emphasizing that a higher percentage of nurses with a master's degree was employed in the university hospital than other hospitals (75.1 vs. 42.3%; χ^2^ = 41.04; *p* < 0.0001). Nurses with a master's degree were younger than those who had lower education level [median 37 (32; 48) years vs. 43 (33; 51) years; *Z* = −2.43; *p* = 0.0147].

### 3.2. Nurses' knowledge of delirium

Nurses' knowledge of delirium varied from 3/37 points to 30/37 points—the median of overall knowledge was 16 (13; 20) points. There was significant difference in median of overall knowledge between female and male nurses [17 (13; 20) vs. 14 (12; 18); *Z* = 2.93; *p* = 0.003]. Respondents with a master's degree in nursing presented significantly higher scores in terms of knowledge of delirium than those who had a bachelor's degree in nursing and registered nurses [median 18 (14; 21) vs. 15 (12; 19); *Z* = 4.71; *p* < 0.0001]. Furthermore, nurses who were employed at university hospital showed higher knowledge scores than those employed in other hospitals [median 18 (14; 21) vs. 15 (12; 18); *Z* = −4.64; *p* < 0.0001]. A weak negative correlation was found between the knowledge and age (rho: −0.17; *t* = −3.41; *p* = 0007), and positive between the knowledge and the number of beds in ICU (rho: 0.19; *t* = 3.80; *p* = 0.0002). Job seniority did not turn out to be associated with nurses' knowledge (*p* > 0.05).

The knowledge deficit concerned the definition of delirium—only 52.3% (*n* = 194) of responders knew that rapid disorientation, change in mental state, disorganized thinking and altered level of consciousness is a definition of delirium. Nurses are not aware that delirium is associated with higher mortality rates−56.9% (*n* = 211) had knowledge on this subject. The least correct answers were given by responders to questions about risk factors for delirium such as: “Hearing impairment increases the risk of delirium” (*n* = 91; 24.5%), “Dementia is the greatest risk factor for delirium” (*n* = 93; 25.1%), “A patient having a repair of a fractured neck of femur has the same risk for delirium as a patient having an elective hip replacement” (*n* = 100; 26.9%), “Diabetes is a high risk factor for delirium” (*n* = 104;28.0%), “A patient with impaired vision is at increased risk of delirium” (*n* = 100; 28.6%) (Table 2).

**Table 2:** Nurses' knowledge of delirium.

**Statement**	**Correct answers**
^**^Fluctuation between orientation and disorientation is not typical of delirium	184 (49.6)
^*^Symptoms of depression may mimic delirium.	156 (42.0)
^**^Treatment for delirium always includes sedation.	224 (60.4)
^**^Patients never remember episodes of delirium	160 (43.2)
^**^A Mini Mental Status Examination (MMSE) is the best way to diagnose delirium.	140 (37.7)
^**^A patient having a repair of a fractured neck of femur has the same risk for delirium as a patient having an elective hip replacement.	100 (26.9)
^**^Delirium never lasts for more than a few hours.	288 (77.6)
^*^The risk for delirium increases with age.	175 (47.2)
^*^A patient with impaired vision is at increased risk of delirium.	106 (28.6)
^*^The greater the number of medications a patient is taking, the greater their risk of delirium	191 (51.5)
^**^A urinary catheter *in situ* reduces the risk of delirium	277 (74.7)
^**^Gender has no effect on the development of delirium	140 (37.7)
^*^Poor nutrition increases the risk of delirium.	209 (56.3)
^*^Dementia is the greatest risk factor for delirium.	93 (25.1)
^*^Males are more at risk for delirium than females.	153 (41.2)
^**^Diabetes is a high-risk factor for delirium.	104 (28.0)
^*^Dehydration can be a risk factor for delirium.	259 (69.8)
^*^Hearing impairment increases the risk of delirium.	91 (24.5)
^**^Obesity is a risk factor for delirium.	196 (52.8)
^**^A patient who is lethargic and difficult to rouse does not have a delirium.	211 (56.9)
^**^Patients with delirium are always physically and/or verbally aggressive.	185 (49.9)
^**^Delirium is generally caused by alcohol withdrawal.	109 (29.4)
^*^Patients with delirium have a higher mortality rate.	211 (56.9)
^**^A family history of dementia predisposes a patient to delirium.	133 (35.8)
^*^Behavioral changes in the course of the day are typical of delirium.	203 (54.7)
^*^A patient with delirium is likely to be easily distracted and/or have difficulty following a conversation.	300 (80.9)
^*^Patients with delirium will often experience perceptual disturbances.	298 (80.3)
^*^Altered sleep/wake cycle may be a symptom of delirium.	233 (62.8)

* I agree.

** I disagree; Results presented as absolute numbers (percentages).

The study subjects lacked knowledge regarding to tools used to assess delirium such as CAM (*n* = 205; 55.3%). The nurses had relatively better knowledge of the use of the AWS scale (*n* = 261; 70.3%), and very good of the use of the DRS scale (*n* = 342; 92.2%).

#### 3.2.1. Determinants of nurses' knowledge of delirium

Factors identified on multivariable analysis (Table 3) as significant determinants of better knowledge of delirium (positive regression coefficients) included master's degree in nursing (vs. Registered nurses + Bachelor's), female gender, and working in university hospital (vs. other). However, nurses' age negatively correlated with their knowledge. This model explained 12% of variance in nurses' knowledge. There was no association between the number of beds in a ward and knowledge of delirium (*p* > 0.05).

**Table 3:** Multiple linear regression analysis for variables predicting for nurses' knowledge of delirium.

**Factors**	**Simple regression ß (95% CI)**	**Multiple regression** **ß (95% Cl)**	**Partial *R*^2^**
Master of Science in Nursing; reference category: Registered Nurse + bachelor's in nursing	0.25 (0.15–0.35)^**^	0.19 (0.08–0.29)^**^	0.04
Female gender	0.17 (0.07–0.27)^*^	0.16 (0.07–0.26)^**^	0.03
Age	−0.17 (−0.27 to −0.06)^*^	−0.14 (−0.24 to −0.04)^*^	0.02
University hospital; reference category: municipal + provincial + other	0.24 (0.14–0.34)^**^	0.13 (0.02–0.31)^*^	0.01
Number of beds in a ward	0.16 (0.06–0.28)^*^	Model *R*^2^ = 0.12, *F*_(4, 365)_ = 13.77; *p* < 0.0001	

ß, standardized regression coefficient; Cl, confidence interval; R^2^, adjusted coefficient of determination.

* p < 0.05.

** p < 0.001.

The assumptions for calculating multiple regression were met: test White'a: p = 0.68; test Shapiro-Wilka: p = 0.35, the Variance Inflation Factor < 1.3.

### 3.3. Clinical practices concerning delirium

One hundred and thirty-one (35.3%) of the total responders acknowledged that the wards where they work have sedation protocols/guidelines, but only one in three responders in this group (*n* = 42) declared that the protocol specified the frequency with that delirium should be assessed. Only 61 (16.4%) nurses declared that they assess delirium often or always, while 133 (35.8%) responders do so rarely and 177 (47.7%) never. It should be noted that assessment of sedation is a common practice in ICUs—only 14.3% (*n* = 53) of responders never measured this parameter. Nurses assessed the presence of symptoms indicative of delirium based on: the patient's ability to follow commands (*n* = 194; 52.3%), relating events (*n* = 172; 46.4%), using the CAM scale (*n* = 89; 24%) or CIWA (*n* = 77; 20.7%), and the screening checklist (*n* = 64; 17.2%). Occasionally, consultations were provided by a psychiatrist (*n* = 133; 35.8%)—one in nine nurses surveyed confirmed that a psychiatric consultation had taken place once during her 12-h duty period. There was no significant association between any of the above elements of clinical practice regarding delirium and the type of hospital and education of the nurses surveyed (*p* > 0.05).

### 3.4. Barriers to effective control of delirium

Table 4 shows the barriers to adequate delirium control in the ICU (the lower the mean and median the more important the barrier). The most significant barrier in the nurses' opinion was the lack of requirement for delirium screening.

**Table 4:** Barriers to effective nurses' control of delirium.

**Statement**	***M***	**Me** **(Q_25_; Q_75_)**
Nurses are not required to screen for delirium in my ICU.	1.5	1 (1; 2)
Difficult to interpret in intubated patients.	1.7	1 (1; 2)
Do not feel confident in my ability to use delirium assessment tools.	1.8	2 (1; 2)
Inability to adequately document delirium assessments.	1.8	2 (1; 2)
Inability to complete assessment in the sedated patient.	1.8	2 (1; 2)
Not enough time to perform assessment (too time consuming).	1.9	2 (1; 3)
Physicians already complete delirium assessments.	2.0	2 (1; 3)
Do not feel that using delirium assessment tool improves outcome.	2.1	2 (2; 3)
Delirium assessment tools are too complex to use.	2.2	2 (2; 3)

Results presented as mean and median (upper and lower quartile).

### 3.5. Nurses' education of delirium

53.1% (*n* = 197) of nurses had never been educated in delirium control. Registered nurses and bachelor's degree nurses were more often not educated than those with a master's degree (63.5 vs. 45.6%; χ^2^ = 11.60; *p* = 0.0007). Only one in five nurses (20.7%; *n* = 77) had the opportunity to learn about delirium during their first and second degree nursing studies. Only 10.5% (*n* = 39) were able to learn about delirium through hospital procedures and/or by attending in-hospital education. Twenty-four percent (*n* = 89) of responders had used other forms of training, with a higher proportion of nurses with a master's degree than colleagues with less education (27.9 vs. 18.6%; χ^2^ = 4.30; *p* = 0.38). The type of hospital where the nurses were employed was not related to delirium education (*p* > 0.05).

### 3.6. Nurses' perception of delirium and delirium care

The study showed that the assessment of delirium in ICU patients is marginalized by nursing staff. When indicating the order of the parameters assessed in these patients, nurses felt that assessing the level of consciousness [median 2 (1; 3)] and pain [median 2 (1; 3)] were the most important, followed by assessing for the presence of agitation [median 3 (2; 3)] and delirium [median 3 (1; 4)]. The least important part of the assessment was to check that invasive devices were placed correctly [median 4 (2; 5)].

**Figure 1:**
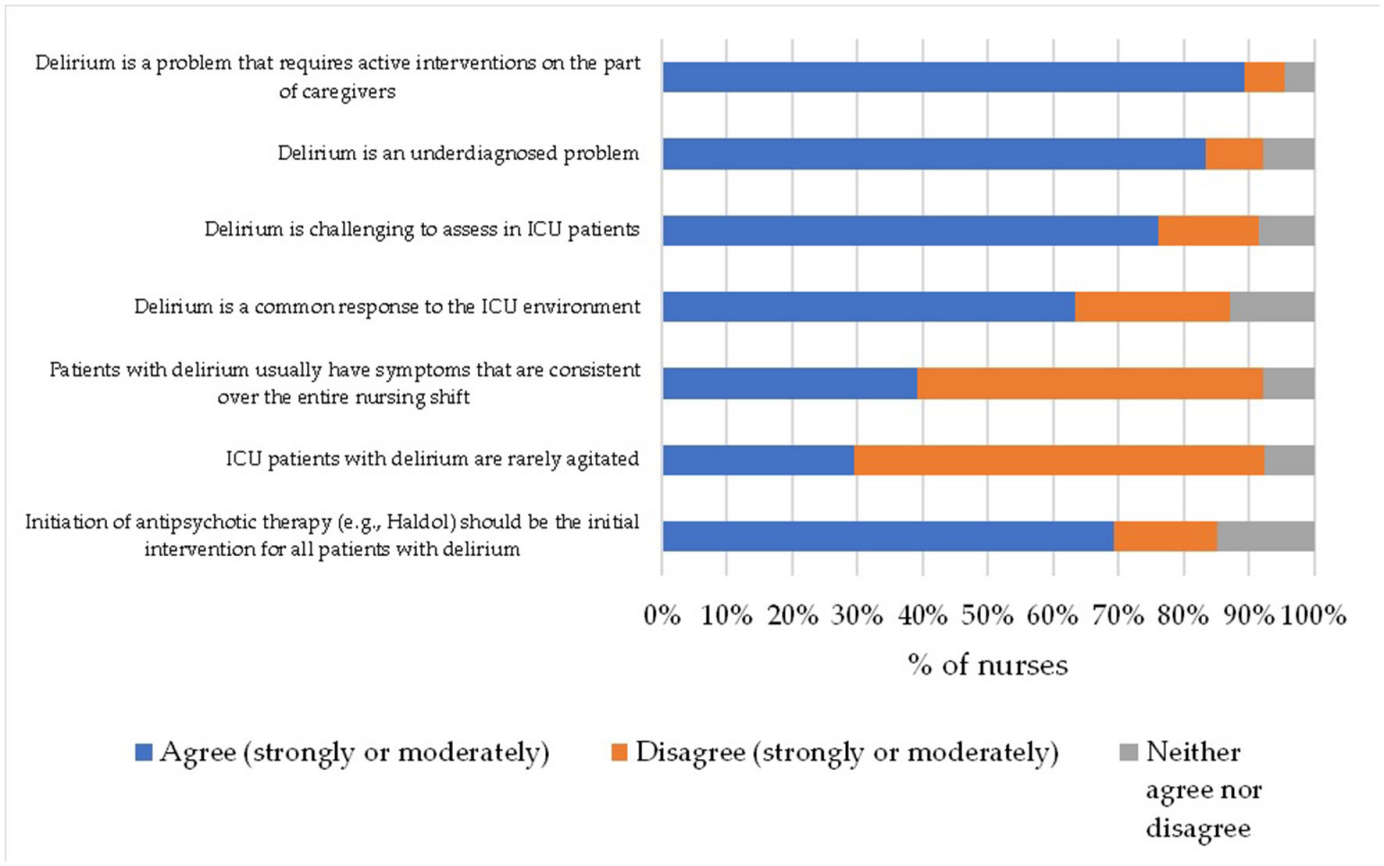
ICU nurses' perception of delirium.

Figure 1 shows the perception of the ICU nurses regarding delirium. More than 80% of them rightly believed that delirium is an under-recognized problem in the ICU and requires active intervention by medical staff. Unfortunately, an equally high percentage of nurses wrongly perceive that antipsychotic treatment should be the first intervention for all patients with delirium (about 80%), probably because, in the nurses' opinion, these patients are most often agitated (70.6%).

## 4. Discussion

The results of the study showed that nurses caring for adult patients in Polish ICUs have a large knowledge deficit in delirium control and do not follow good clinical practice in this area. The lack of obligation to assess delirium is the most important barrier to its adequate monitoring.

### 4.1. Nurses' knowledge of delirium

Despite the increasing interest, available guidelines, and recommendations for the assessment of delirium in ICUs, delirium still remains a marginalized condition and often undiagnosed or misinterpreted by medical staff. It is important to highlight the fact that the prevention and early detection of delirium is key to improving the safety and outcome of ICU patients and provides an opportunity to implement appropriate pharmacological and non-pharmacological interventions to reduce its adverse effects (14). Due to their almost continuous presence and contact with the ICU patient, nurses are the right persons to manage delirium. However, as the study shows, nurses do not have enough knowledge on the topic.

In our study, nurses had difficulty both defining delirium and identifying risk factors. Only 52.3% knew that acute confusion, fluctuating mental state, disorganized thinking, altered level of consciousness. were characteristics that could indicate the development of delirium in ICU patients. In contrast, only one in four nurses were aware that dementia was the most important risk factor for delirium. Nurses were also unaware that delirium was associated with higher mortality. The results may suggest that the majority of ICU nurses might be unable to either correctly identify patients at higher risk of delirium or implement appropriate non-pharmacological interventions that may reduce the risk of developing it (e.g., by providing hearing aids and reading glasses). Furthermore, the responders had no knowledge of tools to assess delirium, such as CAM. Which, in turn, may suggest that staff are not adequately educated on the diagnosis of delirium using the relevant tools. Our results are consistent with a study by Elliott et al. of medical staff in three UK hospitals, in which 42% did not know that delirium in the intensive care unit was associated with higher 6-month mortality, and a high level of knowledge of the definition of delirium was demonstrated by 67% of nursing staff (20).

In addition, our study showed that the determinants that positively influenced knowledge levels were master's degree education, female gender, and employment in university hospitals. Similar relationships between knowledge and gender are reported by Hamdan-Mansour et al. (22). However, the authors cited above do not find a statistically significant association between knowledge and age of nurses (22), in contrast to our study in which we noted a negative correlation between these variables. Perhaps this correlation is due to the different education system in Poland or the easier accessibility of younger nurses to up-to-date knowledge using medical databases and EBM (Evidence-Based Medicine). As in our study, the results obtained by Rowley-Conwy, showed that higher levels of education were also associated with higher levels of knowledge (29).

Given these results, there is a need to disseminate educational materials on delirium to ICU nurses and to create a group of specialists who would be responsible for implementing and promoting recommendations on prevention and care of ICU patients with delirium and educating medical staff. We also recommend the organization of regular conferences, webinars, which will be an effective way to promote and update knowledge about delirium.

### 4.2. Clinical practices concerning delirium

Although assessment of sedation is quite common in ICUs, few of these protocols specify the frequency with which delirium should be assessed. Compared to a study from 2016 conducted by Kotfis et al. among Polish ICU heads, slightly more nurses declared that they assess delirium in their patients often or always (16.4 and 10.9%) (18). Almost half of the responders reported that they never assess delirium in their ICU patients. The results of the Devlin et al. study, among American ICU nurses, also showed that significantly more nurses routinely assess sedation than delirium (98 vs. 47%) (25). In a study by Özsaban et al. among Turkish nurses, it was shown that routine assessment of delirium is performed by 67.8% of ICU nurses (30). Sedation/delirium assessment practices in the above countries are much more common than in Poland. These results are worrying due to the fact that, since 2016, despite the availability of educational materials translated into Polish, validation, and adaptation of delirium assessment scales to Polish conditions, delirium monitoring still remains at a very low level. Comparable results were obtained in a study by Glynn et al. conducted among ICU nurses from Ireland. 19.9% said that their sedation protocol specifies the frequency with which delirium should be assessed, and only 17.9% of nurses reported conducting delirium screening in the ICU (31).

In our study, nurses' assessment of delirium was most often based on the patient's ability to follow instructions (52.3%), recounting events (46.4%). Similar to our study, Irish ICU nurses also indicated that they most frequently assessed delirium based on the patient's ability to follow commands and through agitated related events (37.1%) (31). This is consistent with the results of a study by Devlin et al. in which the preferred method of assessing delirium was also based on ability to follow commands (78%) (25). Use of the validated CAM-ICU tool, in our study, was reported by only 24% of respondents. These results are consistent with those obtained by Özsaban et al. who found that only 14.7% of Turkish ICU nurses used a validated tool to assess delirium (30), while in the Rowley-Conwy study, 38.7% used the CAM-ICU to assess delirium (29). This may indicate that, despite the availability of tools such as CAM-ICU or NuDESC PL, which have been recognized as reliable for use in Poland, there is still a low prevalence of delirium assessment tools. This may be due to inadequate education. It is important to highlight the fact that the non-use of dedicated tools for delirium assessment, may result in a high rate of unrecognized delirium, in particular the hypoactive subtype.

### 4.3. Barriers to effective control of delirium

The most significant barriers, in the assessment of delirium, identified in our study according to nurses were: “In my intensive care unit, nurses are not required to screen for delirium,” “Delirium is difficult to interpret in intubated patients,” and “I do not feel confident in my skills in using delirium assessment tools”. Difficulty in assessing delirium in intubated patients is a common barrier reported by nurses in other studies (32). For example, in the Devlin et al. study, 38% of responders considered the intubated patient to be a barrier, in the Özsaban et al. study the percentage was 66.1%, and in the Rowley-Conwy et al. 58.1% (25, 29, 30). Similarly, in the Scott et al. study, which assessed the effectiveness and feasibility of the CAM-ICU tool before and after delirium education and practical training, intubated patients continued to be the most commonly reported barrier (44 vs. 42.5%) (33). Uncertainty about their ability to use tools to assess delirium is also a barrier reported by nurses in another study (34). This may be due to a lack of knowledge of delirium assessment tools and a lack of training in their practical use. One barrier resulting from the working environment revealed in our study was the lack of obligation for nurses to perform delirium assessments. This highlights the need to implement clear policies and procedures for delirium assessment in ICUs. On the opposite of Devlin et al.'s study, the least important barrier in our study was found to be “The delirium assessment tools are too complex to use”. This result should be interpreted with some caution because it may be due to a lack of common use of delirium assessment tools and not a real perception that they are easy to use.

### 4.4. Nurses' education of delirium

Despite, the high prevalence of delirium among critically ill patients, more than half of the nurses had never been educated in delirium control. Only one in five nurses had the opportunity to gain experience about delirium during their nursing studies. Few nurses were able to learn about delirium through hospital procedures and/or by attending in-hospital education. This is in line with a study by Devlin et al. which found that nurses received little or no education about delirium and this mainly took place in university lectures rather than as practical education at the bedside (25). Similarly, in other studies, the majority of nurses never received any education about delirium (20, 29). Although the theme of delirium is addressed during university education of nurses, the results may suggest that insufficiently. Moreover, the results obtained once again support the need for a change in ward policy regarding delirium and the need for management to implement training programs for ICU staff. In the Scott et al. study, it was shown that after delirium training and practical training in the use of CAM-ICU, nursing staff awareness of delirium and its negative impact on patient outcomes increased (33).

### 4.5. Nurses' perception of delirium and delirium care

Our study showed that the assessment of delirium in ICU patients is a state marginalized by nursing staff. Less important than the assessment of delirium appeared to be only checking that invasive devices are placed properly. According to the nurses, the most important are the assessment of the level of consciousness and the assessment of pain. These results are similar to those obtained by Devlin et al. Among the conditions that nurses considered important for routine care were altered level of consciousness (44%), presence of pain (23%) in first place. Routine assessment of delirium, in the above study, was considered least important (3%) (25). The above results may indicate a lack of awareness among nursing staff that delirium is a state that occurs acutely and that systematic assessment enables its early detection and the implementation of appropriate interventions. The majority of nurses (89%) from our study rightly believe that delirium is an underrecognized problem in the ICU and requires active intervention from caregivers. An equally high proportion of nurses believe that antipsychotic treatment should be the first intervention for all patients with delirium (around 80%). Wynikać to moze z faktu, ze w opinii pielegniarek pacjenci ci sa najcześciej pobudzeni (70.6%). Although, in fact, studies suggest that hyperactive delirium in which patients are agitated is relatively rare in ICU patients. Similar views were expressed by nurses in the Devlin et al. study, who disagreed that patients with delirium are rarely agitated and that initiating antipsychotic treatment (e.g., Haloperidol) should be the first intervention in all patients with delirium (25). This may again be due to a lack of education about delirium, its subtypes, symptoms, and the use of both pharmacological and non-pharmacological interventions. As suggested by the results from our study, which reported a statistically significant correlation between perceptions of delirium and knowledge of delirium and the number of beds in the unit.

## 5. Conclusions

Polish ICU nurses have a knowledge deficit on delirium, and most of them have never had any education on the topic. Moreover, practices in monitoring and assessing delirium are not in compliance with international recommendations. Delirium is a condition marginalized by nurses in ICU patients and is still not routinely assessed in ICUs, and validated tools are not used by nurses. This study also revealed some barriers to the above and may identify areas for improvement in current delirium practices. Firstly, nurses in their units are not required to assess delirium. A clear policy and procedures for delirium management in ICUs would therefore need to be developed and implemented. Intubated patients and nurses' lack of confidence in their ability to use delirium assessment tools are also barriers to delirium assessment. This demonstrates the need to implement educational programs that include both theoretical and practical training at the patient's bedside.

## 6. Limitations

Our study has several limitations that need to be considered. Firstly, data were obtained from all provinces in Poland, unfortunately the fact that single responses were collected from some provinces remains a limitation. Therefore, the results cannot be generalized, but they do provide some insight into current data on delirium care in the ICU and target areas for change. Second, the survey was voluntary in nature; therefore, most people who were interested in the topic of delirium were able to participate in the survey. Thirdly, the survey was anonymous and a survey questionnaire was used as the tool. Therefore, a certain responder bias must be assumed that the results obtained may be overestimated compared to real practice.

## 7. Implications for practice

Due to the deficit in nurses' knowledge of delirium and the significant discrepancy between practice and international recommendations in delirium management, it would be advisable to implement educational programs in ICUs that include both theoretical knowledge and practical training in the use of validated scales at the patient's bedside. In addition, hospital policies and the creation of procedures based on international recommendations for the monitoring and management of delirium in ICUs also need to be changed.

## Data availability statement

The original contributions presented in the study are included in the article/supplementary material, further inquiries can be directed to the corresponding author.

## Ethics statement

The studies involving human participants were reviewed and approved by Independent BioEthical Committee for Scientific Research of the Medical University of Gdansk. The patients/participants provided their written informed consent to participate in this study.

## Author contributions

Conceptualization, methodology, and visualization: SL and WM-D. Formal analysis: LT and SL. Writing original draft preparation: SL, WM-D, and LT. Writing—review and editing: SL, WM-D, LT, and SK. Supervision: MW. All authors have read and agreed to the published version of the manuscript.
